# Emerging Fabrication Strategies of Hydrogels and Its Applications

**DOI:** 10.3390/gels8040205

**Published:** 2022-03-24

**Authors:** Fayaz Ali, Imran Khan, Jianmin Chen, Kalsoom Akhtar, Esraa M. Bakhsh, Sher Bahadar Khan

**Affiliations:** 1 Department of Chemistry, King Abdulaziz University, P.O. Box 80203, Jeddah 21589, Saudi Arabia; fayazalisabir@gmail.com (F.A.); kaskhan@kau.edu.sa (K.A.); ibakhah@kau.edu.sa (E.M.B.); 2 Centre of Excellence for Advance Materials Research, King Abdulaziz University, P.O. Box 80203, Jeddah 21589, Saudi Arabia; 3 State Key Laboratory of Quality Research in Chinese Medicine, Macau University of Science & Technology Avenida Wai Long, Taipa, Macau 999078, China; rustamkhan31@yahoo.com; 4 School of Pharmacy and Medical Technology, Putian University, No. 1133 Xueyuan Zhong Jie, Putian 351100, China

**Keywords:** hydrogels, natural polymers, drug delivery

## Abstract

Recently, hydrogels have been investigated for the controlled release of bioactive molecules, such as for living cell encapsulation and matrices. Due to their remote controllability and quick response, hydrogels are widely used for various applications, including drug delivery. The rate and extent to which the drugs reach their targets are highly dependent on the carriers used in drug delivery systems; therefore the demand for biodegradable and intelligent carriers is progressively increasing. The biodegradable nature of hydrogel has created much interest for its use in drug delivery systems. The first part of this review focuses on emerging fabrication strategies of hydrogel, including physical and chemical cross-linking, as well as radiation cross-linking. The second part describes the applications of hydrogels in various fields, including drug delivery systems. In the end, an overview of the application of hydrogels prepared from several natural polymers in drug delivery is presented.

## 1. Introduction

Hydrogels are the primary interest of this review. Basically, hydrogels are three-dimensional network structures that are prepared from specific natural and synthetic polymers. Hydrogels are defined in many different ways by different scientists. The most common of these is that a hydrogel is a water-swollen and cross-linked polymeric network produced by the simple reaction of one or more monomers. Another definition is that hydrogels have the ability to extensively take in water in their polymer chains and carry sufficient amounts of water in their porous structures, but will not dissolve in water. The hydrophilic functional groups attached to the polymeric backbone provide the ability to absorb water to a higher extent, while the presence of cross-links between network chains provides resistance to dissolution. Therefore, many materials, both naturally occurring and synthetic, fit the definition of hydrogels.

Research on hydrogels was started in the early 1960s by Wichterle and Lim, who published a novel paper on poly (2-hydroxyethyl methacrylate) [1]. Due to their exceptional promises of wide-ranging applicability, hydrogels have received considerable attention in the past 50 years. Hydrogels are under research to explore the fundamentals of swollen polymer networks, because of their ability to absorb water, and they also have wide application in many technical areas, such as in materials for contact lenses and protein separation, devices for the controlled release of proteins and drugs, and dies for encapsulating cells. In practice, water-soluble synthetic polymers are commonly used to achieve high degrees of swelling. Hydrogels may be synthesized in a number of ‘‘classical’’ chemical ways. These include one-step procedures (e.g., polymerization and parallel cross-linking of multifunctional monomers), multiple-step procedures (e.g., synthesis of polymer molecules having reactive groups and their subsequent cross-linking), and polymer reactions with suitable cross-linking agents. Enormous attention is given to the parameters that control degradation characteristics. It is of great importance that the hydrogels used have excellent biocompatibility, and the degradation products have low toxicity. Furthermore, the elastic and soft nature of gels minimizes irritability to the neighboring tissues. Briefly, hydrogels show excellent biocompatibility, and their water-attracting surface has a lower propensity for cells and proteins to stick to it. Based on these properties, hydrogels are an ideal candidate to be applied in biomedical applications, such as wound dressing, controlled drug delivery, tissue engineering, dental materials, cell therapy, sanitary pads, as well as in ophthalmic applications, agriculture, moistures, and sensors [2,3,4].

## 2. Classification of Hydrogels

**Figure 1:**
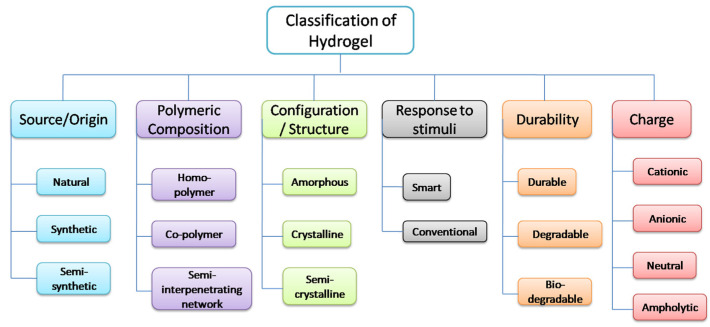
Classification of hydrogels.

Hydrogels can be classified on the basis of source/origin, polymeric composition, structure/configuration, response to stimuli, durability, network electrical charge, and on the basis of cross-linking. These classifications are given in Figure 1, and discussed one by one in the following.

### 2.1. On the Basis of Source/Origin

Hydrogels can be classified into two groups on the basis of source/origin: either natural or synthetic. Natural hydrogels can be obtained from natural sources such as plants and animals [5], while synthetic hydrogels can be prepared from synthetic polymer, i.e., from vinyl monomers by conventional polymerization [6]. Natural hydrogels are gradually being replaced by synthetic hydrogels because of their higher durability, strength, and capacity for water absorption. In addition, synthetic polymers usually have well-defined structures, which can be modified to yield durable, tailorable degradability and functionality. Hydrogels that are prepared from purely synthetic components are also stable in conditions of extreme temperature fluctuations. Recently, dendritic macromolecules or hyperbranched fragments have been reported as an emerging group of synthetic hydrogels with very promising biomedical applications [4,7]. Dendritic macromolecules are well-defined, highly branched macromolecules synthesized via a divergent or convergent approach. A salient feature of the macromolecules described herein, and a focus of our research effort, is the suitability of dendritic macromolecules for in vitro and in vivo use, which can be induced by focusing on biocompatible building blocks and biodegradable linkages. These dendritic macromolecules can be subsequently cross-linked to form hydrogels using a photochemical acrylate-based or a chemical ligation strategy [4,7].

### 2.2. On the Basis of Polymeric Composition

Hydrogels can be categorized into three classes, according to the preparation method and polymeric composition. They are homo-polymeric hydrogels, copolymeric hydrogels and multipolymer interpenetrating polymeric hydrogels (IPN). The methods of preparation are different for each of these important classes of hydrogels:

(a) Homo-polymeric hydrogels are made of one type of monomer as their building block [8]. Based on the nature of the monomer and the polymerization technique, the homo-polymers may have a cross-linked skeletal structure;
(b) Copolymeric hydrogels consist of two or more different monomers having at least one hydrophilic component [9]. These hydrophilic groups are arranged in a block or alternating configuration along the chain of the polymer network, or in a random fashion;
(c) Multipolymer interpenetrating polymeric hydrogels are an important class of hydrogels, made of two independent cross-linked polymer chains [10]. These polymers are either synthetic or natural, enclosed in a network structure. However, one component is a non-cross-linked polymer, and the other component is a cross-linked polymer in semi-IPN hydrogel.

### 2.3. On the Basis of Configuration or Structure

On the basis of the physical structure and chemical composition, hydrogels can be classified into the following three forms:

(a) Crystalline. For example, monodisperse spheres composed of a copolymer of poly(N-isopropylacrylamide) (PNIPAM) and N-hydroxymethylacrylamide (NMA) have been prepared and used as building blocks for a thin hydrogel film. A diluted dispersion of these microgels was allowed to dry in air, resulting in the formation of crystalline microgel structures at the air/dispersion interface [11];
(b) Amorphous (non-crystalline). For example, amorphous hydrogels of carboxymethylcellulose [12];
(c) Semi-crystalline. These are a complex mixture of amorphous and crystalline phases. For example, semi-crystalline poly (vinyl alcohol) hydrogels [13].

### 2.4. On the Basis of Response to Stimuli

The characteristics of hydrogels, such as swelling and de-swelling, depend on the surrounding environment. Therefore, hydrogels are sensitive to stimuli and respond to the features of the external environment, such as the presence of electrolytes, pH and temperature. Hydrogels experience a change of phase or volume collapse in response to various physical or chemical stimuli. Hydrogels that are temperature-sensitive are also called thermogels [14]. Novel pH-sensitive, physically cross-linked hydrogels were synthesized by grafting D,L-lactic acid (LA) onto the amino groups in chitosan (CS) without a catalyst by Xin Qu et al. [15]. When the polymers are shrunk by cooling below the upper critical solution temperature, these types of hydrogels may exhibit positive thermo-sensitive swelling. Stimuli-responsive hydrogels include poly (N-isopropyl acrylamide) gels, poly (vinyl methyl ether), etc. [16,17].

### 2.5. On the Basis of Durability

Hydrogels can be classified into two main categories on the basis of durability, as either durable or bio-degradable. These properties are relevant to their specific applications.

#### 2.5.1. Durable

Generally, synthetic hydrogels are durable. For example, pristine silk fibroin (SF) hydrogels with excellent mechanical properties were fabricated by Zhu [18], using a binary solvent-induced conformation transition (BSICT) strategy.

#### 2.5.2. Bio-Degradable

Generally, natural hydrogels are bio-degradable. For instance, the in vivo biocompatibility of a poly (2-hydroxy-ethyl-L-glutamine) biodegradable hydrogel was examined using a cage implant system [19]. Further, degradable polymers may be classified on the basis of the nature of bond breaking. Sensitive bonds can be broken due to either enzymatic or hydrolytic actions.

### 2.6. On the Basis of Network Electrical Charge

On the basis of the presence or absence of electrical charge on the cross-linked chain, hydrogels may be categorized into four groups:

(a) Ionic (including anionic or cationic)—Ionization in gel formation leads to the development of fixed charges, and thus the forming of anionic or cationic hydrogels. Acidic pendant groups form anionic hydrogels, and consequently, ionization takes place when the pH of the environment is higher than the pKa of the ionized group. The degree of ionization increases while the pH increases, and this raises the quantity of fixed charges. In this case, the increase in pH causes electrostatic repulsions and thus increased swelling. The basic pendant groups comprise cationic hydrogels, such as amines. These groups undergo ionization at a pKb lower than that of the ionizable groups. Here, the decrease in pH increases the electrostatic repulsion and causes increased swelling. For instance, poly (acrylic acid) (PAA) is used as a highly ionic conductive hydrogel [20];
(b) Nonionic (neutral)—These hydrogels have permanent linkages in the polymer network, which are irreversible. These hydrogels swell or de-swell in response to changes in temperature. Poly (*N*-isopropylacrylamide) (PNIPA) is an example of a non-ionic hydrogel [21];
(c) Zwitterionic (polybetaines)—These hydrogels are also known as polybetaines. Monomers of these hydrogels contain both anionic and cationic groups. Poly (sulfobetaine methacrylate) (polySBMA) hydrogels have been reported as zwitterionic hydrogels [22];
(d) Amphoteric electrolyte (ampholytic)—These hydrogels contain monomers of both acidic and basic groups. The properties of these gels are dependent on the ionic groups attached to the chains. They can be attracted to solutions bearing an opposite charge, and thus, they may show either inter-ionic or intra-ionic interactions. The pH-sensitive swelling of a natural–polyelectrolyte complex gel, prepared from xanthan and chitosan, was investigated using a model based on the Donnan equilibrium theory, with special attention paid to the dissociation behavior of the polyelectrolytes [23].

### 2.7. On the Basis of the Type of Cross-Linking

Based on the physical or chemical nature of the cross-link junctions, hydrogels can be divided into two categories. Physical networks have transient junctions that arise from either physical interaction, such as hydrogen bonds, ionic interactions and hydrophobic interactions, or from polymer chain entanglements, while chemically cross-linked networks have permanent junctions. 

## 3. Emerging Fabrication Strategies of Hydrogels

Generally, three basic components (monomer, initiator and cross-linker) are involved in the fabrication of hydrogels. These components must be present in such a ratio that the integrity and consistency of the gel does not transform. The hydrogels are thoroughly washed with ethanol or water in the last step of hydrogel preparation to remove all unreacted initiator, cross-linker and monomer. Various natural and synthetic methods are used to develop hydrogels, such as homo-polymeric and copolymeric methods. Only one type of monomer is involved with cross-linking in the homo-polymeric method. Various monomers are used in the preparation of these types of hydrogels, including carbohydrates, lipids, nucleic acids and proteins. Basically, monomers bind chemically to the second molecules in order to form a polymer—an example of this is the hydrogel prepared from polyacrylic acid (PAA). Polysaccharide-based hydrogels are being intensively investigated, because it is very easy to derive gelatin from them, and they possess excellent biological activities [24]. Due to these properties, polysaccharides, such as microcrystalline cellulose, and their derivatives are being prepared for cephalexin delivery [25]. Similarly, dendritic macromolecules can be subsequently cross-linked to form hydrogels using a photochemical acrylate-based or a chemical ligation strategy. The properties—mechanical, swelling, degradation, and so forth—of the hydrogels can be tuned by altering the composition, cross-linking chemistry, wt. %, generation number and so on [7].

In contrast, copolymeric hydrogels consist of two monomers, one of which should be hydrophilic. Copolymeric hydrogels are developing using various mechanisms. Generally, two monomers are cross-linked with the help of a cross-linking agent, and then the initiator is added separately. Such types of hydrogels are synthesized and discuss in the given references [26,27]. 

### 3.1. Hydrogel Preparation by Cross-Linking Method

Hydrogels are prepared using cross-linking techniques, such as adapted physical, chemical or radiation cross-linking, and by grafting polymerization. The viscoelasticity and mechanical properties relevant to biomedical applications and pharmaceutical fields can be improved by such modifications. The general methods of producing hydrogels using the above-mentioned cross-linking methods are described below.

#### 3.1.1. Physical Cross-Linking

The advantage of using no cross-linking agent, and their relative easy production, make physical or reversible gels more interesting as compared to others. Currently, this area is receiving considerable attention because a broad range of gel textures can be formed by the careful selection of hydrocolloid type, pH, and concentration. In the literature, physically cross-linked hydrogels have been created using various methods, which are explained here one by one.

##### Heating/Cooling a Polymer Solution

A heating–cooling photopolymerization process was applied to prepare double-network hydrogels recently [28]. The researchers used agar, N-benzylacrylamide (NBAA) and N-acryloyl glycinamide (NAGA) monomer in a single water pot in the presence of UV initiators. In another study, a synthetic polymer model system was used based on polyisocyanides, which were cross-linked inside a bundle [29]. Rheology and X-ray have shown that the network’s mechanics can be accurately tailored by tuning its thermal history, and not by changing the gel composition or architecture. Hydrogel is formed via helix formation. For instance, carrageenan appears as a random coil transformation in hot solutions above the melting transition temperature, and upon cooling it forms rigid helical rods. Double helices further aggregate to form stable hydrogels in the presence of salt (Na^+^, K^+^, etc.) because of the repulsion of the sulfonic group [30]. Examples are polyethylene glycol-polyactic acid and polyethylene oxide-polypropylene oxide. 

##### Ionic Interaction

Ionic interaction is also a type of physical cross-linking; for instance, alginate may be cross-linked at room temperature and physiological pH via calcium ions [31]. Alginate gels may be used for living cell encapsulation [32] and as a matrix for protein release [33]. Ionic and covalent cross-linkers are used to prepared chitosan-coupled ionic/covalent hydrogels [34]. The separate contributions of ionic and chemical cross-linking have been investigated, and it has been found that ionic cross-linking is disturbed by the addition of genipin to the neutralized solution, as a result of which chemical cross-linking has become the dominant approach [34]. The addition of di- or tri-valent counter ions of ionic polymers can help cross-link these polymers. Gelling via this method is exemplified by hydrogel formation from a polyelectrolyte solution (e.g., Na^+^ alginate^−^) with a multivalent ion of opposite charges (e.g., Ca^2+^ + 2Cl^−^) [2,35,36].

##### H-Bonding

Hydrogen bonding occurs in complexes of polymethacrylic acid and polyacrylic acid, with polyethylene glycol between the carboxylic group of polymethacrylic acid/polyacrylic acid and the oxygen of the polyethylene glycol [37]. Hydrogen bonding is also found in poly (methacrylic acid-g-ethylene glycol) [38,39]. The protonation of carboxylic acid gives rise to hydrogen bonds, which cause swelling in the pH-dependent gels. Recently, a double-network hydrogel mainly cross-linked by a hydrogen bond was reported by Li et al. [40]. The first network is made of gelatin, and the other is a poly(N-hydroxyethyl acrylamide) network. The resultant hydrogels show fast self-recovery at room temperature, high mechanical properties, and good self-healing properties. Similarly, in another study, a supermolecular hydrogel formed from multiple hydrogen bond interactions was reported, which exhibited robust, self-healing and shape-memorizing properties (high-speed recovery) [28]. These double network hydrogels show excellent biocompatibility and extensive applications in biomedical materials.

##### By Protein Interaction

Protein engineering is a new field in material chemistry that was pioneered by Tirrell and Cappello [41,42]. The peptide sequence is the main advantage of protein engineering, as a result of which physical and chemical properties may be controlled in synthetic DNA sequences. Synthetic amino acids may also be used in addition to natural amino acids [43]. Through genetic engineering, sequential blocks containing elastin-like and silk-like block repetitions from synthesized polymer were prepared by Cappello and co-workers [42,44]. Polyacrylamide was grafted to rabbit IgG in the presence of an additional cross-linking agent, i.e., antibody [45]. The hydrogel showed slight swelling in the presence of free antigen due to the polymer-bound antigen replacement, which resulted in a reduction in cross-linking density along with the release of antibodies.

#### 3.1.2. Chemical Cross-Linking

Recently, because of its good mechanical strength, increased interest has been shown in chemically cross-linked hydrogels. Different methods have been applied in the literature to synthesize chemically cross-linked hydrogels, as given below.

##### Chemical Cross-Linkers

Hydrogel development is possible with the help of hydrophilic groups, namely, NH_2_, COOH and OH, which are present in hydrophilic polymers. Covalent linkages between polymer chains may be recognized in these reactions by the formation of a Schiff base, or an isocyanate–OH/NH_2_ or amine–carboxylic acid formation. Examples of chemical cross-linkers are given in Table 1, and are discussed in the following paragraphs.

##### Cross-Linking by Addition Reaction

Hydrophilic polymer functional groups react with Bis or higher functional cross-linkers during addition reactions. Polysaccharides may be cross-linked by means of 1,6-hexanedibromide [46], divinylsulfone [47], or 1,6 examethylenediisocyanate.

##### Cross-Linking with Aldehydes

-OH groups containing hydrophilic polymers may be cross-linked through aldehyde, for example, polyvinyl alcohol can be linked through glutaraldehyde [48]. Strict conditions are applied to establish cross-linking, such as low pH, high temperature, and methanol addition as a quencher. Alternatively, amine group polymers cross-linked under mild conditions using the same cross-linker, and Schiff bases are formed. Cross-linked proteins can be synthesized by special designs, such as gelatin [49], amine containing-polysaccharides [50], and others mentioned in the review article by Faheem et al. [51].

##### Cross-Linking by Condensation Reactions

Condensation reactions of the –NH_2_ or –OH groups with –COOH or derivatives are carried out to synthesize polyamides and polyesters, respectively. Ray and co-workers reported hydrogel synthesis using these reactions [52]. Fejin and co-workers synthesized gelatin hydrogel using N,N-(3-dimethylaminopropyl)-N-ethyl carbodiimide (EDC), which is an efficient hydrophilic polymer reagent for cross-linking an amide group [53]. N-hydroxysuccinimide was added during the reaction to achieve superior hydrogel cross-linking density and restrict any side reaction. Moreover, anionic polysaccharide (chondroitin sulfate) was also added into the hydrogel to improve the loading capacity [54].

**Table 1:** Some examples of chemically cross-linked hydrogels.

S. No	Polymer	Method Type	Loaded Drug	References
1	Chitosan	Cross-linking with aldehyde	Indomethacin	[55]
2	Pectin/chitosan	Cross-linking by Diels-Alder reaction	5-Fluorouracil	[56]
3	Gelatin	Cross-linking with aldehyde	TGF-β1	[49]
4	Chitosan	Cross-linking with aldehyde	Mitoxantrone	[50]
5	Dextran	Addition reaction	Hydrocortisome	[57]
6	Gelatin	Condensation reaction	Lysoszyme	[53]
7	PVA	Condensation reaction	Diltiazem hydrochloride	[52]
8	Albumin	Cross-linking with aldehyde	Adriamycin	[58]
9	Chitosan–PVA	Cross-linking with aldehyde	Nano-insulin	[48]

##### Cross-Linking by Free Radical Polymerization

Besides the free radical polymerization of vinyl monomers mixtures, chemically cross-linked hydrogels may also be fabricated from the polymerizable group of hydrophilic polymers by free radical polymerization. Natural, semi-synthetic and synthetic hydrophilic polymers have been used to prepare gels using this method. Hydrogel is also synthesized with the help of UV polymerization. A photo-reversible system is also possible, under which hydrogels start to degrade after exposure to UV light, which releases the drug [59].

##### Cross-Linking by High Radiation

A chemical cross-linked hydrogel may be formed with the use of high-energy radiation. In the literature, high radiation sources are reported to polymerize unsaturated substances, e.g., electron beam/thermal [60] and gamma rays [61]. Recently, gamma radiation has been used as an initiator to fabricate superabsorbent hydrogel using acrylic acid-co-vinyl acetate and agar/gelatin, with N-N′ dimethylenebisacrylamide as a cross-linker. The swelling behavior was investigated in distilled water, and the maximum Ps achieved was 8010 as a function of temperature, pH and time [62]. In another study, a chitosan-based pH-responsive amphiphilic terpolymer hydrogel was synthesized using gamma radiation for colon cancer drug delivery. This hydrogel has the ability to release Fluorouracil up to 96% at pH 7 after 7 h [63]. Moreover, pH-sensitive hydrogels copolymerized by a graft polymerization method (gamma rays) were designed by Moises and co-workers, for the localized prophylactic release of silver nanoparticles (AgNPs) and ciprofloxacin for topical bacterial infections [64]. A detailed study of the synthesis of hyaluronic acid-polyvinyl alcohol hydrogel by gamma irradiation for biomedical applications can be found in a book recently published [65]. Ultraviolet (UV) radiation is also used for this purpose; poly (ethylene oxide) (PEO) with a molecular weight from 200,000 to 2,000,000 was cross-linked by exposure to ultraviolet radiation with a high-pressure 150 W mercury lamp [66]. Similarly, PEO modified with a quaternary tetraalkyl ammonium salt, ethyl methacrylate dimethyldodecylammonium bromide, was prepared by exposure of the solid blend to ultraviolet radiation using a high-pressure 150 W mercury lamp [67]. It was reported that when the UV cross-linking was performed in the frozen state, porous hydrogels with very high yields of gel fraction (above 90%) and high cross-linking density were obtained. After drying the hydrogels, films of 50–150 μm thickness were prepared. These films swell extremely fast in water and act as asymmetric membranes [68].

##### Cross-Linking Using Enzymes

Enzymatically cross-linked PEG-based hydrogels were prepared by Sperinde and co-workers [69]. They observed that in the gel, the diffusion coefficients of albumin and small proteins are comparable to those in free solution. Under mild conditions, gelation occurs, and these gels form highly hydrated networks around living cells. Moreover, Nicole and co-workers enzymatically cross-linked silk fibroin and hyaluronic acid (HA) to form hydrogels with tunable mechanical properties similar to those of native tissues [70]. Injectable hydrogels were synthesized by an in situ forming method using the enzymatic cross-linking of polymer–phenol conjugates in the presence of hydrogen peroxide (H_2_O_2_) and horseradish peroxide (HRP) to improve adhesion properties [71]. Similarly, in the presence of HRP and H_2_O_2_, injectable hydrogels were developed under physiological conditions using poly(L-glutamic acid) grafted with tyramine, and poly(ethylene glycol) was enzymatically cross-linked, which has biomedical applications, including in drug delivery carriers and tissue engineering scaffolds [72]. A microfluidic device based on an enzymatically cross-linkable gelatin hydrogel system was developed by Samantha and co-workers [73] to explore the effects of human astrocyte reactivity and hypoxia-induced oxidative stress on rat neuroglia and myelin production. An artificial ovary is a promising approach for preserving fertility. An artificial ovary helps to maintain the proliferation and ovarian stromal cell viability. Based on silk fibroin- (SF) and phenol-conjugated chitosan, through an enzymatic cross-linking method, a double-network hydrogel using HRP was developed with a high water swelling capacity [70]. Recently, an enzymatically cross-linked tyramine–gellan gum hydrogel as a drug delivery system for rheumatoid arthritis (RA) treatment was developed by Isabel and co-workers [74]. In addition, for osteochondral tissue engineering, a reinforced hydrogel with dual-lineage bioactivity was developed from enzymatically cross-linked silk–nanosilicate [75]. Furthermore, cartilage repair was also achieved with the help of enzymatically cross-linked natural polymers, as reported by Zoetebeir [76]. They used dextrin and hyaluronic acid to prepare enzymatically cross-linked hydrogels for the regeneration of damaged cartilage.

##### Cross-Linking by Grafting

Generally, hydrogels fabricated with the use of bulk polymerization have weak mechanical properties and structures. Hydrogels may be grafted onto a stronger support to improve the above-mentioned characteristics. Under this grafting technique, a variety of polymeric supports have been used for the synthesis of hydrogel. An example of this kind of process is starch grafted with acrylic acid using N-vinyl-2-pyrrolidone [77].

### 3.2. Magnetic Hydrogel Preparation

Magnetic particles in hydrogels generate the characteristic of magnetic response. Therefore, the crucial step in magnetic hydrogel preparation is the addition of magnetic particles into the hydrogel network. Magnetic hydrogel performance mainly depends on type, size and concentration of gel and MNPs, as well as the distribution of nanoparticles in the hydrogels. Generally, blending, in situ and grafting-onto methods are used to fabricate magnetic hydrogels. Magnetic hydrogels can be synthesized from both natural and synthetic polymers using the above-mentioned method. However, the grafting-onto method is the least applicable for hydrogel preparation using natural polymers because of the lack of active sites. Therefore, the blending and in situ methods are commonly used in the preparation of magnetic natural hydrogel, but some are fabricated by the grafting-onto method. These preparation processes for magnetic hydrogel are explained in the following, along with their advantages and disadvantages.

#### 3.2.1. Blending Method

Blending is a commonly used method for the preparation of magnetic hydrogel because of its convenience and simplicity. Generally, this method is used to blend metal nanoparticles (MNPs) in a gel. In this method, MNPs need to be prepared separately before blending in the polymer solution. Usually, sonication is used to obtain well-dispersed MNPs in a polymer solution. The MNPs encapsulated in the polymer form the magnetic hydrogel by virtue of using different cross-linking agents. Due to its simplicity, this method can be widely applied. However, there are some limitations to using this method, such as the leakage of MNPs when the hydrogel is exposed to a liquid environment, as well as the aggregation of MNPs.

Previously, this method has been used to fabricate several polymer hydrogels. For example, magnetic gelatin nanoparticles were prepared by encapsulating Fe_3_O_4_ nanoparticles in gelatin, and then dispersing these nanoparticles in oxidized alginate and carboxyethyl chitosan solutions to form hydrogels for biomedical applications [78]. Similarly, in another study, magnetic cellulose hydrogels were synthesized by dropwise addition to a CaCL_2_ coagulation bath of ultrasonically dispersed Fe_3_O_4_/β-cyclodextrin/cellulose mixture [79]. Inorganic substances may also be used as fillers to form magnetic hydrogel by combining with magnetic Fe_3_O_4_ nanoparticles, as reported by Dai et al. [80]. Significant potential in broad applications has been shown by the above-mentioned preparation methods; however, during blending, the uneven distribution of MNPs in the polymer network may hinder their further development. MNPs’ distribution in the magnetic hydrogel can be improved by loading Fe_3_O_4_ onto the nanofibrillated cellulose before mixing with the polymer solution. Preparing stable nanocomposites containing MNPs using this strategy is expected to be useful method.

#### 3.2.2. In Situ Method

Another convenient and practical procedure for magnetic hydrogel preparation is in situ method. In this method, MNPs are introduce after the formation of hydrogel; for instance, a hydrogel is first formed from the polymer solution by cross-linking and then immersing in Fe^2+/^Fe^3+^ solution, so these ions can spread throughout the existing network of hydrogels. Finally, the loaded hydrogels are dipped in precipitating agents, such as NH_3_·H_2_O and NaOH. As compared to the blending method, this method is easy to operate, and the MNPs are well dispersed in the hydrogel network. In addition, the strong interaction of the polymer and Fe_3_O_4_ can prevent the escape of MNPs from hydrogels [81]. However, this method is only applicable for stable hydrogels that are not destroyed by alkali solutions. Moreover, due to the use of alkali solutions in these magnetic hydrogel preparations, they are not suitable for cell encapsulation. Additionally, a complex may be formed from Fe^2+/^Fe^3+^ in the presence of negatively charged functional ligands, and thus a low yield of Fe_3_O_4_ hydrogel may be obtained [82]. Besides this, copper oxide-antimony oxide (Cu_2_O-Sb_2_O_3_) was prepared and entrapped inside a Na-alginate hydrogel (Alg@Cu_2_O-Sb_2_O_3_) [83,84,85,86].

The in situ method is considered an ideal tool for the preparation of magnetic hydrogels, and cellulose polymer is the most commonly used natural polymer under this method [87]. The magnetic natural polymer hydrogels synthesized by the in situ method may have weak mechanical properties, which hinder their usability in different applications. Therefore, the gel strength could be improved by blending the natural polymer with poly (vinyl alcohol) (PVA) to fabricate high-strength hybrid hydrogels. By repeating the freeze–thawing method, the PVA can form strong hydrogen bonds with natural polymers. At present, the freeze–thawing method is commonly used to develop PVA/cellulose and PVA/chitin magnetic hydrogels, which are further decorated in situ with Fe_3_O_4_ [88,89]. Moreover, several properties have been improved by adding nanocellulose in the preparation of hydrogels; for instance, swelling ability, thermal stability and drug loading. However, mechanical properties were not studied [90]. In future, the synergistic effect of PVA and the effect of nanocellulose on the strength of magnetic hydrogels should be considered.

#### 3.2.3. Grafting-Onto Method

The grafting-onto method is rarely used, as compared to the blending and in situ methods, in the fabrication of natural polymer magnetic hydrogels. Besides few differences, this method is similar to the blending method. In this method, functional groups are grafted onto MNPs before they are mixed with polymer solution. These grafted MNPs can work as cross-linking agents, and thus there is no need for adding other cross-linkers. The sizes of MNPs are uniform, and they are well dispersed and stable in the polymer matrix. However, the fabrication method is time-consuming, high-cost and complex. Hyaluronic acid/carboxymethyl cellulose magnetic hydrogels were synthesized by this method [91]. The researchers used (3-aminopropyl) trimethoxysilane to functionalize the prepared MNPs (CoFe_2_O_3_ and Fe_3_O_4_). The amine groups of functionalized MNPs work as cross-linkers, and thus form amide bonds with carboxylic groups of carboxymethyl cellulose. The fabricated hydrogels were suitable for remotely controlled drug delivery via a magnetic field.

## 4. Application of Hydrogels

**Figure 2:**
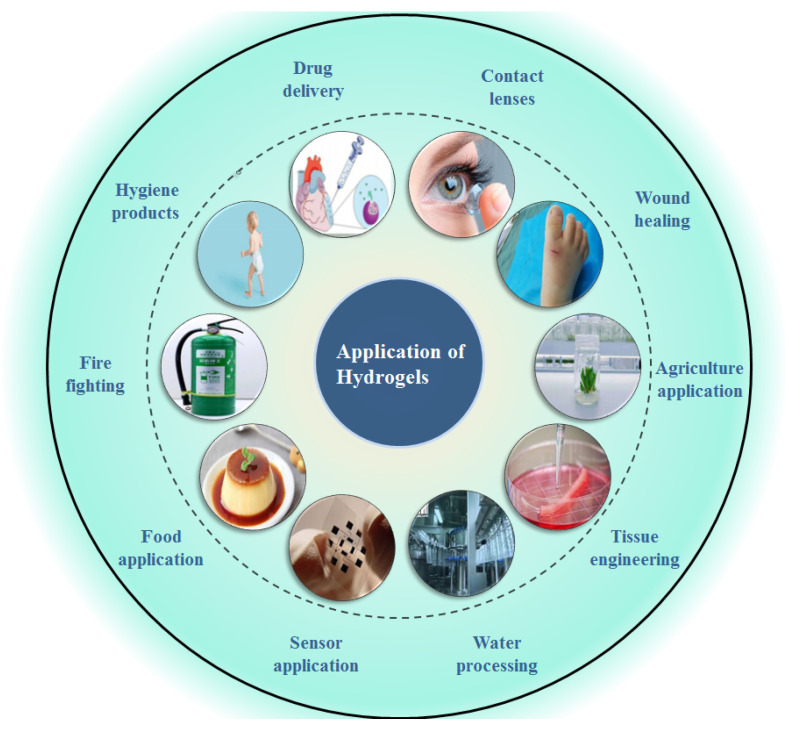
Hydrogels have been applied in all aspects of human life.

Due to the advantages of hydrogels over other types of biomaterials, including their good swelling behavior and mechanical strength, increased biocompatibility, tunable biodegradability and low toxicity, they have been widely used in the fields of wound healing, tissue engineering, water processing, agricultural application, food applications, sensor applications, firefighting and other applications [92] (absorbent hygiene products and contact lenses), as shown in Figure 2. Besides this, they have also been broadly applied in the field of drug delivery, which will be discussed separately in the next section. In this section, the recent advances in the use of hydrogels in the aforementioned applications will be reviewed.

### 4.1. Wound Healing

Wound healing (also known as the regeneration of wounds) is a dynamic biological process that can be classification into four continuous processes, including hemostasis, inflammation, proliferation and remodeling [93]. These processes are quite complicated and affected by various factors, which may cause abnormal wound repair [94]. Therefore, wound dressings are commonly employed to cover the wound surface and provide additional functionalities that can speed up the healing processes [95]. Due to the excellent biocompatibility, biodegradability, hydrophilicity, comfortability and functionality of hydrogels, they are the most promising candidates for wound dressings, and have garnered particular attention from many researchers [96]. The original application of hydrogels in wound healing only focused on simple physical isolation and creating a moist environment, but with the development of the research, more and more novel hydrogels have been designed and prepared with various functions, such as anti-bacterial, anti-oxidant, anti-inflammatory and stimulus responsivity [97]. In this part, we will discuss the recent advances in functional hydrogels as wound dressings for wound healing.

It is well known that bacterial infection is the greatest challenge to the wound healing process. Therefore, much attention has been paid to the development of anti-bacterial hydrogels, and these can be generally divided into three categories, including anti-bacterial agent-containing hydrogels, inorganic nanoparticle-containing hydrogels, and hydrogels with inherent anti-bacterial capabilities [98]. Anti-bacterial agent-containing hydrogels are the most popular type for wound dressings, and many anti-microbial drugs have been incorporated into hydrogels, such as chlorhexidine acetate [99], ampicillin [100], sulfadiazine [101], ciprofloxacin [102], doxycycline [103] and simvastatin [104]. Inorganic nanoparticles such as silver [105], gold [106], zinc [107] and copper [108] are well known for their usability as anti-bacterial materials, and have also been used to prepare anti-bacterial hydrogels for would healing. However, some potential risks of long-term retention and biotoxicity associated with the application of inorganic materials should be addressed firstly. The drug resistance of anti-bacterial agents and the biological toxicity of anti-bacterial inorganic materials increase the demand for better anti-microbial hydrogels. Therefore, hydrogels with inherent anti-bacterial capabilities have been proposed recently. These hydrogels are usually composed of natural or synthetic anti-bacterial polymers, including chitosan [109,110], modified chitosan [111], polyethylenimine [112], peptides [113] and pseudoprotein [114].

The wound healing process sometimes induces excessive inflammation, which may result in high oxidative stress and a significant increase in the production of reactive oxygen species (ROS). ROS, such as hydroxyl radicals, hydrogen peroxide and superoxide anions, may cause oxidative damages to biological macromolecules such as proteins and DNA [115]. This has prompted many scientists to develop strategies to scavenge ROS and reduce oxidative injury, and a promising measure is the use of anti-oxidants that can trap and neutralize ROS [116]. Therefore, the incorporation anti-oxidants into hydrogels can significantly enhance wound healing. Anti-oxidants including natural flavonoids [117], polyphenols [118], anthocyanins [119], resveratrol [120], dopamine [121], gallic acid [122] and red jujube extract [116] have been encapsulated into hydrogels for their ability to scavenge ROS and promote wound healing. Furthermore, the incorporation of anti-inflammatory agents such as quercetin [123], curcumin [124], sericin [125], superoxide dismutase (SOD) [126] and prostaglandin E2 [127] into hydrogels will directly prevent the occurrence of excessive inflammation, which may help to promote wound healing, especially for chronic wounds.

Stimuli-responsive hydrogels are sensitive to external environmental factors, such as temperature, pH, light, electricity and magnetic fields, and can make appropriate changes to their morphology and structure [128]. Due to their unique properties, they have broad application prospects in the field of wound healing. For example, thermally responsive hydrogel precursor solutions (N-isopropylacrylamide monomer, NIPAM) can flow at low temperatures, and can be easily injected into a wound; subsequently, the solutions are rapidly transformed into hydrogel at a physiological temperature [129]. This makes the application of hydrogels easy, and simplifies the therapeutic process of wound healing. Therefore, PNIPAM-based thermos-responsive hydrogels have attracted much attention from scientists recently [130,131,132]. pH-responsive hydrogels are prepared by a reduction in the coordination between tannic acid and metal ions (forming a Schiff base structure), which can be dissociated under acidic conditions and thus release insulin, which will help the healing of diabetic wounds [133]. Similarly, light-responsive hydrogels can also release some drugs in a controlled fashion when they are stimulated by near-infrared light [102]. Electricity-responsive hydrogels, containing the conductive component polyaniline, have been proven to better promote the recovery of wounds when compared with hydrogels without polyaniline [134]. Additionally, it has been demonstrated that the incorporation of magnetic nanoparticles into hydrogels also enhances wound healing [135].

### 4.2. Tissue Engineering

Tissue engineering (TE) is an interdisciplinary scientific field that integrates life sciences with engineering in order to develop substitute tissues or promote tissue repairs [136]. The scaffold, cell and growth factor are the critical components of TE, which are associated with the construction and regeneration of tissues [137]. The scaffold can mimic the native extracellular matrix (ECM) and provide a suitable physiological environment for the adhesion, differentiation, migration and proliferation of the seeding cells [137]. Owing to the excellent biocompatibility, biodegradability, non-toxicity and flexibility of hydrogels, they have become the most promising scaffold materials for TE [138]. It is well known that hydrogels have been widely used in TE, such as in bone, cartilage, nerve and cardiac tissues.

Bone and cartilage tissues are both skeletal tissues, which are closely related to the movement of the human body [139]. It brings a lot of inconvenience into people’s lives if skeletal tissues are damaged. Therefore, hydrogels for bone and cartilage TE have been paid much attention in recent years. It has been reported that the incorporation of hydroxyapatite (HAP) nanoparticles into hydrogels makes the materials for bone TE more flexible and robust [140]. Besides this, a more bioactive mineral, tricalcium phosphate, has been used to replace HAP for bone tissue regeneration, since it is more readily degradable [141]. Natural cartilage has high stiffness and tensile strength properties, so common hydrogels cannot meet the requirement for mechanical properties [142]. This encourages scientists to combine stiff nanomaterials with soft polymers to form nanocomposite hydrogels, the mechanical properties of which have been greatly improved [143,144].

Previous studies have demonstrated that electrical stimulation could induce cellular responses, especially in nerve and cardiac cells [145]. Therefore, to repair nerve and cardiac tissues, multifunctional hydrogels, especially those with electrical properties, have been developed recently [146]. Directly incorporating conductive nanomaterials into hydrogels is the most convenient strategy to fabricate nanocomposite hydrogels with high conductivity. For instance, carbon nanotube [147], grapheme [148], polypyrrole [149] and polyurethane [150] have all been confirmed to improve the electricity of hydrogels, and promote the neuronal differentiation of neural stem cells; thus, they can be used as materials for nerve tissue repairing. Cardiovascular disease is the number one cause of death worldwide, and the regenerability of cardiac tissues is limited. Therefore, cardiac TE has been considered as a potent strategy for cardiac regeneration. Metal-based nanomaterials, such as gold nanoparticles [151], titanium dioxide nanofiller [152], and glass nanoparticles [153] with excellent biodegradability, porosity and electricity have been incorporated into hydrogels, and then applied to replace or repair injured cardiac tissue.

### 4.3. Water Processing

People take it for granted that water will always be plentiful, since it covers 70% of our planet. Actually, only 3% of the world’s water is fresh water, which we drink, bathe in and irrigate our farm fields with. With the rapid development of modern societies and the global economy, water resources are becoming increasingly valuable. Researchers all over the world have developed various methods to obtain clean, fresh water in order to solve the problem of freshwater reduction [154]. Hydrogel-based methods play a key role in tackling this problem, and have been widely used in water processing, including water evaporation, water desalination, and wastewater remediation.

Hydrogel-based materials play an important role in the process of water evaporation, which is the main method used to obtain clean fresh water. It is very convenient and environmentally friendly to use solar energy for water evaporation, which can be applied in sterilization, water treatment, wastewater remediation, desalination and steam generation [154]. Thus, photothermal conversion materials are essential components of hydrogels, and can increase solar absorption. For example, copper sulfide–microporous polyacrylamide [155], reduced graphene oxide [156], titanium sesquioxide [157] and carbon black nanoparticles [158] were all used to achieve the high optical absorbance of solar radiation, and thus improve solar water evaporation. In the specific case of water desalination, some factors of hydrogels need to be taken into consideration, including hydrophilicity for fluid transport, high photothermal conversion efficiency, and a large specific surface area for evaporation [154]. All this means that the materials incorporated into hydrogels should be able to improve the photothermal energy conversion. For instance, a carbon-based hybrid hydrogel was applied in a water desalination process, and the researchers obtained a photothermal conversion efficiency of 94.5% [159].

The discharge of wastewater, especially dye-containing wastewater, causes the destruction of the aquatic environment and is harmful to the human body [160]. Specifically, the disposal of colored effluents from the food industry poses a challenge to the environment, since food dyes are generally nonbiodegradable. In order to remove dye contaminants from water for environmental protection, a number of methods, such as adsorption, precipitation, photocatalytic degradation, biological treatment and chemical oxidation, have been proposed [161]. Adsorption is one of the most effective and economical methods of tackling with dye pollution, when compared with the more common chemical and physical dye removal methods. Due to the three dimensional (3D) network structures and excellent hydrophilicity of hydrogels, they have become promising materials for dye removal [162]. It was reported that a carboxymethyl cellulose hydrogel embedded with modified magnetite nanoparticles and porous carbon can be used as a dye adsorbent, and shows great potential in the treatment of wastewater [163]. Additionally, the incorporation of organic fibrils, clay minerals, carbonaceous materials and magnetic particles into pure hydrogels was carried out to produce composite hydrogels, which have been proposed to remediate dye-contaminated water [164].

### 4.4. Agriculture Application

Agriculture associated with the production of essential food crops plays an essential role in the entire life of any economy. Additionally, it also provides employment opportunities to a very large percentage of the population, and thus is essential to a nation. However, currently, agriculture is being restricted by some key factors, such as limited water resources, imbalanced fertilizer application, low soil organic carbon and pest and disease control [165]. Due to their excellent ability to absorb huge amounts of water, hydrogels are also being used in agriculture, mainly for saving irrigation water, achieving the sustained release of fertilizers, and improving soil quality and pesticide management [166].

As mentioned above, water scarcity is a global challenge, and thus water is a major constraining factor in crop production. Therefore, some technologies have been proposed to enhance the water utilization ratio in agricultural product production. Among these technologies, super-absorbent hydrogels (water-saving materials) have been widely applied in agriculture in advanced countries [167]. Hydrogels have 3D hydrophilic networks that can absorb and retain huge amounts of water. It was demonstrated that the water utilization ratio and irrigation intervals can be increased by the application of these hydrogels [168]. It was confirmed that these hydrogels have a significant effect on plant height, and can increase grain yield [169,170]. Therefore, the application of hydrogels alleviates the adverse effects of inadequate irrigation and drought stress conditions on plant growth.

Fertilizer is one of the most important ingredients used to improve soil fertility, which has a critical impact on agriculture. However, conventional fertilizers with high solubility, low thermal stability and small molecular weight are easily lost in surface run-off, denitrification, leaching and volatilization, resulting in low fertilizer use efficiency [171]. Therefore, there has been an increasing interest in developing fertilizer with a sustained release capacity. The combination of hydrogels and fertilizers is proposed and used to obtain the controlled or sustained release of fertilizers, thus improving the fertilizer use efficiency [172]. For example, the sustained release of nitrogen was achieved using hydrogel-based fertilizers composed of polyacrylamide and urea, realizing the long-term release of urea (up to 40 days) [173]. Similarly, a chitosan/starch hydrogel-based fertilizer was prepared to realize the slow release of potassium nitrate by controlling the mass ratio of the chitosan and starch [174].

Soil quality associated with bulk density, water content, nutrient retention and content of heavy metals is important for plant growth and yield. Soil bulk density is a critical parameter in soil quality, and greater than 1.5 or 1.7 g/cm^3^ will restrict the natural root growth, but this can be decreased by the application of hydrogels [175]. The low water-holding capacity and nutrient loss of the soil can be addressed by adding hydrogels [167], cases of which have been discussed above. It has also been demonstrated that the incorporation of hydrogels and biochar into soil can reduce the uptake of heavy metals by potato and spinach plants grown with contaminated wastewater [176]. Similarly, synthesized composite hydrogels have shown great potential for the simultaneous remediation and fertility improvement of heavy metal-contaminated soil [177]. Therefore, hydrogels can promote plant growth and yield by modifying the quality of soil.

Pesticides mainly include herbicides and insecticides. Pesticide application for protecting crops is crucial, and it is necessary to make this process as efficient as possible [178]. Many methods of pesticide management have been developed to improve the utilization rate of pesticides. Hydrogels can be used in pesticide management by providing the sustained and controlled release of pesticide. Atrazine is a reliable, effective and less expensive herbicide, but its easy leaching property limits its application. Therefore, it has been encapsulated into agar/starch/polyacrylamide composite hydrogels and released in a sustained manner [179]. Besides this, a broad range of insecticides, such as organophosphates and carbamate, are being released in a sustained and controlled manner using hydrogels, which can reduce their harmful effects [180].

### 4.5. Food Application

The food industry is a complex network of diverse businesses, which range from farming and food production, to packaging and distribution and retail and catering. Recently, hydrogels prepared for food applications have received considerable attention, as they show great potential in modulating various properties of hydrogel-based food products [181]. They can be used to improve the stability and bioavailability of bioactive food ingredients, serve as fat substitutes, and produce edible or biodegradable food packaging, which will be discussed below.

The organoleptic, anti-bacterial and nutritional characteristics of food products can be improved by incorporating various bioactive compounds, such as probiotics and aroma compounds. However, due to their low stability, bioavailability and solubility, the use of these compounds in food products is restricted [182]. These issues can be solved by the encapsulating technique. Given their excellent properties, including low cost, good biocompatibility and high encapsulation efficiency, using hydrogels as encapsulation materials has gained much attention from scientists [183]. Chitosan-based hydrogels was used to encapsulate curcumin, and the encapsulation efficiency reached 90.3% [184]. In addition, carboxymethyl cellulose-based composite hydrogels showed an encapsulation efficiency of 94.7% in the encapsulation of probiotics, which can be released in a controlled manner [185].

Chronic diseases, such as obesity, diabetes and coronary heart disease, are associated with the high consumption of fat-rich foods. Therefore, it is highly desirable to develop food products with reduced fat, but this will bring about undesirable characteristics in the food, such as hard texture, atypical flavor and decreased water retention [181]. Fortunately, hydrogels made from proteins and/or polysaccharides can partially replace fat droplets, because high water-content hydrogels can retain the textures of foods and produce a similar lubrication effect to full fat products [186]. For example, in the process of producing mayonnaises, 20% of fat was replaced by whey protein–pectin, which possesses desirable rheological properties, such as thixotropy, viscosity and elasticity [187].

The food industry attaches great importance to food packaging materials, since they can reduce the loss of flavor and nutrition and increase the shelf life of food [188]. Traditional biodegradable and edible food packages generally have weak resistance against gases, low mechanical strength, and poor water resistance properties [189]. Therefore, natural polymer-sourced interpenetrating network hydrogels have been used to develop some biodegradable and edible films, which have superior mechanical strength and show excellent resistance to gases, vapors and water [190]. In a recent study, a composite film composed of zein and chitosan exhibited lower oxygen, carbon dioxide and water vapor permeability; this film was used for mushroom packaging and exhibited a lower weight loss rate, respiration rate and relative leakage rate [191]. Besides this, chitosan-based hydrogels with excellent properties (mainly hydrophilicity, flexibility, biodegradability and high permeability) when used as food packaging materials have been used in the preservation of meat, vegetables and fruits [192].

### 4.6. Sensor Application

A sensor can be defined as a device to convert the input of physical, chemical or biological stimuli into a functionally related output, usually in the form of an electrical or optical signal [193]. Therefore, sensors can be used to detect the physical characteristics of objects and the chemical and biological properties of molecules, including pressure, strain, flow, temperature, position, odor, pH, and the presence of special molecules [193]. In recent years, hydrogels have been successfully integrated into various transduction systems to develop sensors, which mainly include pressure and strain sensors, electrochemical sensors and biosensors [194].

Pressure and strain sensors, especially those with the property of flexibility, have shown promising applicability in wearable and implantable devices and artificial skin and soft robotics [195]. Because of the biocompatibility and biomimetic properties of hydrogels, flexible sensors based on hydrogels have greater advantages than other sensors. Pressure sensors are used to transform pressure into other signals, and thus have great potential for applications in artificial intelligence and physiological signal monitoring [196]. Dong et al. have developed a pressure sensor based on ELF-patterned hydrogel surfaces, which increase the contact area and thus improve the sensitivity and precision of dynamic pressure sensing [197]. Another pressure sensor with superior mechanical and sensing properties was developed based on a nature-inspired ionic double-network hydrogel, which is comprised of sodium alginate nanofibrils and polyacrylamide [198]. Strain sensors can transform strain or deformation into electronic signals, which can be used to capture real-time signals from the cardiac tissues, epidermis and joints [199]. Highly stretchable hydrogels composed of carbon nanotubes, silver nanowires and graphene can be used as strain sensors [200]. A composite hydrogel was used to construct strain sensors, which are easily attached to the skin for recording human respiration and pulses [201].

Electrochemical sensors are considered to be very suitable for detecting analyte response, with high selectivity, accuracy and precision [202]. In recent years, composite hydrogels have been successfully integrated with electrochemical transduction devices, which can respond to a variety of analytes. Various hydrogels, especially nanocomposite hydrogels (incorporated with metal nanoparticles and carbon nanomaterials), have been proposed to fabricate electrochemical sensors for the detection of analytes [203]. For example, a polyacrylamide-based hydrogel sensor was fabricated to detect glucose in sweat samples, the contents of which were indicated by the changes in electrical properties (capacitance and impedance) [204]. Besides this, numerous analytes, such as H_2_O_2_, Pb^2+^, fructose, dopamine, paracetamol, hydroquinone, catechol and ascorbic acid, have all been detected by hydrogel-based electrochemical sensors [205].

Biosensors are devices composed of a transducer, a physicochemical detector and a bioreceptor, which are used to detect the presence or concentration of a biological analyte [206]. Bioreceptors play a key role in biosensors, and can detect or identify the target analyte by specific interactions, which impart high selectivity for detection. Hydrogels have been widely applied in the field of biosensors, and are mainly used in the immobilization matrix, responsive unit and wearable device [207]. Using a hydrogel as the immobilization matrix is superior to using other materials in terms of maintaining the biological activity of the biological probe, and the reduction or even prevention of nonspecific binding, because of the highly hydrated structure (similar to biological tissue) and antifouling properties of hydrogels [208]. As an immobilization matrix, a 3D peptide hydrogel was used to immobilize oligonucleotides (bioprobe) for detecting DNA sequences, and this made the oligonucleotides probe less prone to degradation by nucleases [209]. Hydrogels can also be used as responsive units, because they can respond to physical or chemical changes in their environment. For example, urease was immobilized on a hydrogel and catalyzed the degradation of urea, the reaction products of which led to a volumetric change in the hydrogel, indicating the existing of urea [210].

### 4.7. Fire Fighting

Fire accidents always lead to a large loss of wealth, and pose a momentous threat to human life [211]. Therefore, the development of fire-extinguishing agents or fire-resistant materials with good flame retardant properties is crucial to prevent fire accidents. Due to its high heat capacity and latent heat of vaporization, water has been commonly used for fire prevention and extinguishing [212]. However, most of the water is wasted in most cases of firefighting because of the strong fluidity of water, and the improper treatment of the wastewater often causes water pollution [213]. Therefore, the research into and development of new environmentally friendly fire-extinguishing agents has been highly desirable. Recently, hydrogels have attracted great interest as materials protecting humans, buildings and forests from fire.

According to the World Fire Statistics Center, 80,000 people have died and 800,000 people have been injured in fires every year since the start of the 21st century [214]. Most fire accidents happen in confined spaces, such as houses and vehicles, and thus it is very important to develop effective fireproof materials, such as fireproof coatings and blankets for protecting human beings. When hydrogels with a high water content are exposed to a flame, they can keep a low temperature (approximately 100 °C) compared to traditional fire-retardant materials [215]. Therefore, hydrogels have been widely used in the fabrication of novel fireproof coatings and blankets. For example, a new type of fire-retardant material was prepared by laminating a hydrogel and a fabric, and this material can protect the skin from burn injuries, indicating that it is a promising candidate for saving human lives in fire accidents [215].

Wooden buildings are being revived because of global concerns about climate change and natural resource constraints, and thus the growth rate of wooden houses in the UK is faster than that of masonry houses [216]. Due to its advantages, such as renewable nature, easy processing, excellent strength-to-weight ratio and beautiful texture, wood plays a key role both in buildings and in our daily life [217]. However, the inherent flammability of wood and its products limits its wide applicability. Therefore, hydrogels have been used as coatings for wooden buildings and other wood products. For example, gelatin-based hydrogels are used to produce a self-repairing, biodegradable and fully recyclable coating, giving wood superior fire retardant properties [218]. Coated wood can attain excellent self-healing abilities, as well as complete recyclability and fire resistance, without affecting the mechanical properties.

Forest fires occur all over the world every year, and have a destructive impact on the environment. Moreover, forest fires can have a significant impact on human life, including direct property losses and health problems caused by air and water pollution [219]. Compared with conventional fire extinguishers, such as those using nitrogen and water, hydrogels reduce temperature, thermal radiation dose and CO generation by forming an additional layer on flammable materials [220]. In recent years, hydrogels have become widely used in preventing forest fires. A study reported that a multiphase hydrogel was developed to improve fire-extinguishing efficiency [221]. In another study, a silica hydrogel was used as fire-extinguishing material especially for forest fires, the flame retardance of which was 50 times greater than that of ordinary water [222].

### 4.8. Other Applications

In addition to the applications of hydrogels mentioned above, they also have certain application prospects in other aspects, such as the manufacturing of absorbent hygiene products and contact lenses. Absorbent hygiene products, such as baby diapers, feminine hygiene products and adult incontinence products play an essential role in the quality of life and skin health of humans [223]. Compared to traditional absorbent materials such as paper, cotton, cloth and wadding, hydrogels have great advantages in the field of hygiene products, because of their strong capacity to absorb water, their high mechanical strength, and their excellent biocompatibility and biodegradability. For example, hydrogels have been proven to possess the ability to absorb water or biological fluids up to 1000 times their weight [224]. Moreover, hydrogels can keep moisture away from the skin, promote skin health, prevent diaper rush and bacterial colonization, a nd reduce the risk of fecal contamination and the potential spread of gastrointestinal infections [225]. Notably, to avoid the production of solid wastes, completely biodegradable hydrogels based on sodium carboxymethylcellulose and hydroxyethyl cellulose have been developed [226].

Contact lenses are medical devices commonly worn for vision correction, but also used for purely cosmetic purposes. Silicone hydrogel-based contact lenses were launched onto the market by CIBA Vision, marking the most notable progress in the development of contact lenses. Hydrogel-based biomaterials for the production of contact lenses must have specific characteristics, including permeability to oxygen and ions, comfortableness, hydrolytic stability, biological inertness, and the capacity to maintain clear and stable vision [227]. Besides this, the hydrogels used to fabricate contact lenses can transmit at least 90% of visible light, which is essential to obtaining good visual performance [227]. Moreover, in order to improve the comfort of contact lenses, hydrogels have been modified to reduce friction by increasing the lubricity of surfaces [228]. It is noteworthy that the mechanical properties of hydrogels are not easy to measure because of the hydrophilicity of polymers and the large amount of water in the network [229].

Another emerging application of hydrogels is as carriers with a biocatalytic function (immobilization of enzymes). Hydrogels of natural origins and the entrapment method have become increasingly popular in terms of enzyme immobilization. Recently, invertase immobilization using two natural hydrogel matrices—alginate and gelatin—were studied, and the gelatin-based hydrogel was selected as an effective carrier for invertase immobilization [230]. In another study, a mixed-charge nonfouling pseudozwitterionic hydrogel was prepared, and its pH-responsive adsorption shows potential for use in a biocompatible tissue engineering matrix or membrane enzyme reactors [231]. The usage of DNA hydrogels for enzyme entrapment in an enzymatic biobattery has also been reported [232], and the synthesis and application of a new hydrogel based on a methacrylate substituted polyphosphazene was also investigated. The results of this were that enzyme loading reached a maximum of 24.02 mg/g, with activity retention of 67.25% when the methacrylic acid concentration was 20% (*w*/*w*) [233].

## 5. Applications of Hydrogels in Drug Delivery

**Figure 3:**
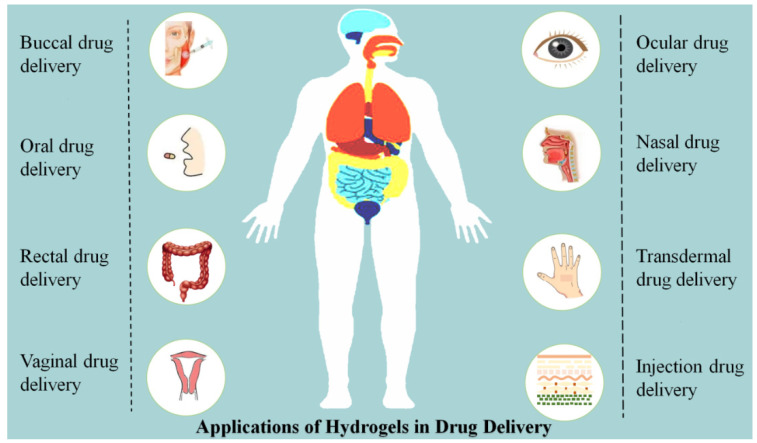
The application of hydrogels in different routes of drug delivery.

As mentioned before, the unique physicochemical properties of hydrogels have attracted special interest for their application in drug delivery. More specifically, the porous structure of hydrogels that can be easily tuned via cross-linking enables the incorporation of drugs into the gel matrix and drug release in a controllable rate, which depends on the diffusion coefficient of the small molecule or macromolecule through the gel network. Besides this, the excellent properties of hydrogels, such as their high biocompatibility, suitable mechanical strength, good biodegradability, high water content and low toxicity, mean they are widely used in various routes of drug delivery, including ocular, nasal, buccal, oral, vaginal, rectal, transdermal, subcutaneous and intramuscular (injection) drug delivery, as shown in Figure 3. In this section, recent advances in the application of hydrogels via different routes for drug delivery have been reviewed and discussed.

### 5.1. Ocular Drug Delivery

The eye is one of the most complicated organs of the body, and is responsible for the visual system. The eye consists of two segments—anterior and posterior—which mainly include the cornea, conjunctiva, aqueous humor, iris, pupil, crystalline lens, retina, macula, optic nerve, choroid, sclera and vitreous [234]. Due to eye diseases such as glaucoma, diabetic retinopathy, conjunctivitis and retinal vascular diseases, visual impairment has resulted in a large number of people worldwide with varying degrees of inability to work [235]. However, the structure of the eye provides natural barriers that help prevent external substances (including drugs) from entering the eye, and it thus presents a great challenge for the ocular delivery of drugs. Therefore, scientists are working to develop novel drug delivery systems to overcome the low bioavailability of conventional ophthalmic formulations. Various hydrogels have been emerging as new materials for the delivery of drugs to eyes.

For example, thermos-responsive hydrogels (also known as temperature-sensitive) have been developed based on poly (N-isopropylacrylamide) [236]. It has been demonstrated that these hydrogels can achieve the localized release of bevacizumab or ranibizumab for about a month, and this does not have long-term effects on retinal function. The controlled biodegradation and complete release of these hydrogels can be achieved by adding biodegradable copolymers and other additives. Another study extended the release of bevacizumab by using novel biodegradable thermos-responsive hydrogels made of poly (2-ethyl-2-oxazoline)-b-poly (ε-caprolactone)-b-poly (2-ethyl-2-oxazoline) [237]. These hydrogels have been demonstrated to have in vitro and in vivo biocompatibility with human retinal pigment epithelial cell lines in a rabbit model over two months. Additionally, a thermo-responsive chitosan/gelatin-based hydrogel used for the sustained release of latanoprost has been proposed, which was proven to significantly decrease intraocular pressure within 7 days after application in rabbit eyes [238].

Hydrogels with pH responsiveness have also been developed for ocular drug delivery. The most common polymers used for preparing such kinds of hydrogels include chitosan, polycarboxyl, carbomer and polyacrylic acid. The polymer solution can flow when the pH value is lower than 7.2 (the pH of tears), but the solution undergoes a gelation process when it comes into contact with the eye [239]. A pH-responsive hydrogel based on sodium carboxymethylcellulose and carbomer have been developed for the ocular delivery of dexamethasone to treat uveitis [240]. The results of in vitro and in vivo studies in rabbit eyes have shown a more significant improvement in the anti-inflammatory activity of the hydrogels than in that of a marketed solution formulation. Moreover, a pH-responsive in situ gelling product (Tiopex^®^), for the delivery of timolol maleate to treat glaucoma, is available on the market [241]. It is noteworthy that the pH of the hydrogels should not exceed the range of pH 4–10, since this may cause irritation or damage to eye tissue [242].

In addition to hydrogels responsive to these stimuli (such as thermal and pH), contact lenses have also been used for ocular drug delivery, because of their unique advantages, such as easy termination of therapy and high bioavailability [243]. For instance, contact lenses modified by vitamin E have been used to deliver various drugs (such as pirfenidone, dorzolamide, timolol and fluconazole) with sustained release and improved drug loading [241]. Besides this, using hydrogels to form microneedles has been proposed to deliver drugs to the eye. It has been reported that hydrogel-formed microneedles were employed to deliver bevacizumab to eyes with sustained and controlled release [244]. Moreover, the same research group proposed a novel process of forming hydrogel microneedles with interlocking features in order to secure the microneedles in place [245].

### 5.2. Nasal Drug Delivery

Nasal drug delivery (known as nasal administration, or snorting) is a route of administration through the nose that can achieve local or systemic administration. The nasal cavity is a good channel for drugs to enter the systemic circulation and central nervous system, because of the high permeability of the nasal mucosa, which is derived from its high vascularity, low thickness and large absorptive surface area [246]. In addition, it also has the potential to carry drugs directly across the blood–brain barrier through olfactory and trigeminal nerve cells [247]. Based on these findings, hydrogel-based formulations have been developed for delivering drugs through the nose for the treatment of local diseases, intranasal vaccinations, the systemic delivery of drugs, and the direct delivery of therapeutic molecules to the brain [248].

Hydrogels can be used for the treatment of local diseases, such as nasal congestion and allergic rhinitis. A thermos-responsive hydrogel encapsulating phenylephrine hydrochloride was prepared to develop a prolonged remedy for nasal congestion [249]. The hydrogel achieved a sustained release of phenylephrine hydrochloride for up to 8 h, and showed a similar toxicity to saline (the negative control), indicating it is a potential formulation that can help achieve prolonged decongestion in the nasal cavity. Allergic rhinitis is a type I allergic inflammatory disease, which is an allergic response of the nasal mucosa to specific allergens. Chitosan hydrogel containing nucleic acid (miRNA-146) was prepared, and its pharmacodynamic effects in allergic rhinitis were evaluated [250]. This hydrogel was able to release miRNA into the mucosa in a sustained manner after nasal administration, and showed better performance in terms of its delivery ability and pharmacodynamic effects than other formulations, which has been further confirmed in ovalbumin-induced rhinitis rats.

The nasal mucosa has been given much attention, since it offers the possibility of obtaining both mucosal and systemic immune responses, and thus it has become an attractive target site for vaccination. Hydrogel particles made of chitosan or composite chitosan were prepared to deliver vaccines across the nasal mucosa, which improved local and systemic immune responses to tetanus toxoid [251,252]. Similarly, chitosan nanoparticles encapsulating plasmid DNA were prepared for nasal mucosal immunization against hepatitis B, which induced both humoral and cellular immune responses [253]. A novel thermal responsive hydrogel composed of modified chitosan was prepared and used to deliver a Zaire Ebola virus glycoprotein antigen to the nasal mucosa [254]. The study demonstrated that this hydrogel-based vaccination induced a high level of antibody titers (IgG) in the serum and mucosal IgA responses in lung wash, and showed low toxicity against nasal tissue and epithelial cells. However, nasal vaccination still has some limitations, such as complicated production procedures, the instability of antigens, low immunogenicity and potential toxicity, which must be addressed to advance the development of nasal vaccine delivery systems.

Nasal drug delivery, especially approaches based on hydrogels for their systemic effects, has attracted much interest, because hydrogels can enhance drug absorption by reducing swallowing and increasing the drug’s retention time in the nasal cavity [255]. Cross-linked chitosan was used to prepare hydrogels for the nasal delivery of insulin, which can enable sustained release (six hours longer than the control) [256]. The application of this hydrogel in delivering insulin through the nose was associated with a significantly prolonged hyperglycemic effect and pharmacological efficiency in diabetic rats. A thermos-responsive hydrogel (based on polyethylene glycol methyl ether) was prepared to encapsulate risedronate for nasal drug delivery [257]. The hydrogel was proven able to achieve the sustained release and augmented permeability of risedronate when compared to other groups. In addition, an ion-responsive hydrogel was proposed to deliver ketorolac tromethamine to the nasal cavity for its systemic effects [258]. The hydrogel was demonstrated to be a potentially useful intranasal analgesic formulation, due to the improved intranasal absorption, sustained drug release, obvious pharmacodynamic effects and negligible nasal toxicity it induced.

Delivering drugs to the central nervous system is a major challenge in the treatment of nervous system diseases, such as Alzheimer’s disease, Parkinson’s disease, schizophrenia and meningitis. The main obstacle to drugs entering the brain after systemic administration is the existence of a membrane barrier called the blood–brain barrier [259]. Nasal drug delivery is of great interest for researchers, because drugs may cross the blood–brain barrier via the olfactory pathway [260]. For example, a thermo-responsive hydrogel (based on pluronic) was developed to load lorazepam for intranasal brain targeting, which helped obtain improved bioavailability and sustained drug release [261]. A hydrogel composed of Poloxamer and carbopol was prepared to deliver naratriptan hydrochloride through the olfactory lobe pathway for migraine headaches [262]. Additionally, it was also demonstrated that hydrogels made of pluronic are a potent carrier for use in the brain-targeting of rivastigmine tartarate via a nasal drug delivery system [263].

### 5.3. Buccal Drug Delivery

The buccal drug delivery involves the delivery of drugs through the buccal mucosa for its local or systemic effects, and it has some advantages, such as avoiding contact with digestive fluids, bypassing the first pass effect, better patient compliance and the option of terminating administration in time. Therefore, buccal drug delivery is a promising research field for the delivery of some special drugs, especially for peptides and proteins. Several dosage forms, including film, patch, tablet, microparticles and hydrogels, have been applied for buccal drug delivery. The advantages of using hydrogels for buccal delivery include extended retention time in the oral cavity, adequate drug penetration, high efficacy and better patient compliance. Generally, mucoadhesive hydrogel films are the most common dosage form, and they be used for the buccal delivery of both hydrophilic and hydrophobic drugs.

Hydrogels are more suitable for the buccal delivery of hydrophilic drugs. A thermo-responsive hydrogel prepared mainly by using Poloxamer analogs was developed for the buccal delivery of salbutamol, which is used to relieve the symptoms of asthma and chronic obstructive pulmonary disease [264]. Another study reported that a hydrogel made of chitosan glutamate was developed for the buccal delivery of lidocaine hydrochloride (an anesthetic drug) [265]. The anesthetic activity of this hydrogel was evaluated in vivo, the results of which suggest the hydrogel found a promising application in the relief of symptoms of aphthosis and other painful mouth diseases. Similarly, a buccal drug delivery system using a novel catechol-functionalized chitosan hydrogel was developed for delivering lidocaine hydrochloride [266]. The catechol groups have been confirmed to significantly enhance the mucoadhesion of hydrogel in vitro, using a porcine mucosal membrane as the model. The hydrogel achieved the sustained release of lidocaine hydrochloride for about 3 h in rabbit buccal mucosa, and no inflammation was observed on the buccal tissue, indicating it has potential applicability in buccal drug delivery. Besides this, other hydrophilic drugs, including metoclopramide, ornidazole, insulin and recombinant human epidermal growth factor, have also been encapsulated in hydrogels prepared by various materials [267].

Several hydrophobic drugs have also been loaded in hydrogels for buccal drug delivery. For example, a chitosan hydrogel prepared by physically (freeze-drying) cross-linking was developed for the buccal delivery of denbufylline, which can be released in a sustained manner over at least 5 h [268]. Ketoprofen, a non-steroidal anti-inflammatory drug, was also encapsulated in a carbopol-based hydrogel, which has been prepared by the photo-polymerization technique for the first time [269]. Another anti-inflammatory agent (naringin) was also encapsulated in a thermos- and pH-responsive hydrogel for buccal drug delivery to inhibit periodontitis [270]. In addition, a mucoadhesive hydrogel was developed by the freeze/thaw cross-linking technique for loading econazole nitrate to treat oral candidiasis [271]. The results show that the sustained release of the drug was achieved, and the growth of candida albicans could be inhibited for 12 h.

### 5.4. Oral Drug Delivery

Oral drug delivery is considered the most preferred and convenient administration route, mainly due to its high patient compliance, ease of administration, and noninvasiveness [272]. The most common dosage forms for oral delivery include tablets, capsules, suspensions, emulsions and powders. However, these dosage forms face significant challenges, such as chances of choking, limited dose flexibility, limited permeation, difficulty dissolving and physiochemical instability [273]. Hydrogels show a promising applicability in oral drug delivery because of their tunable properties, excellent biocompatibility, and release of drugs in a controlled way. Therefore, hydrogels have been extensively studied for the oral delivery of various drugs, including both chemical drugs and biopharmaceuticals.

Hydrogels have been used to encapsulate several chemical drugs, such as diclofenac sodium, curcumin, aspirin, paracetamol, ciprofloxacin, amifostine [274] and magnolol [275], for oral delivery. For example, an alginate-based hydrogel was prepared as an oral drug carrier for the delivery of diclofenac sodium, which can be released in a controlled manner [276]. A pH-responsive composite hydrogel (based on hyaluronic acid/gelatin) containing curcumin was designed and evaluated [277]. The hydrogel exhibited good, sustained release properties, and a high level of curcumin was maintained for more than 24 h. Additionally, a novel acrylic acid-grafted hydrogel was used as an oral drug delivery vehicle for aspirin and paracetamol [278]. A synthesized polymeric hydrogel was employed as an oral delivery carrier for ciprofloxacin [279].

Biopharmaceuticals, including insulin, DNA, probiotics, interferon and some vaccines, have all been delivered orally using hydrogels as vehicles. For instance, an improved food gum-based hydrogel was designed and assessed for the oral delivery of insulin [280]. Insulin was loaded into the hydrogel using a swelling diffusion approach and delivered to diabetic rats orally, which led to a sustained decrease in fasting plasma glucose levels over 6 h. Similarly, another study developed hydrogel microparticles using polysaccharides for oral insulin delivery, and realized the controlled and sustained release of the drug [281]. A study has demonstrated that chitosan-coated DNA hydrogel microspheres could be used to deliver DNA via the oral route [282]. A chemically modified alginate hydrogel was designed for the oral delivery of probiotics [283]. Additionally, hydrogel nanoparticles based on poly (methacrylic acid-grafted-ethylene glycol) were developed and evaluated as an oral drug delivery vehicle for the chemotherapeutic agent interferon [284].

Most commercially available vaccines are still immunized by injection, but oral vaccines have attracted great interest recently because of their relatively enhanced safety, the convenience of their administration, and their ability to induce a mucosal immune response [285]. The potential of using pH-responsive hydrogel microparticles, prepared using bacterial nanocellulose/polyacrylic acid, as an oral vaccine carrier was investigated [286]. A high content (72%) of ovalbumin was encapsulated in the hydrogel, and the drug was released in a pH-dependent manner. The results of in vivo experiments suggest that the oral delivery of an ovalbumin-loaded hydrogel generated significantly higher levels of serum IgG and mucosal IgA than the intramuscular administration of ovalbumin. Furthermore, the same group used these hydrogel carriers for the oral delivery of a hepatitis B antigen, the results of which demonstrated that this antigen-loaded hydrogel can be used as an oral vaccine for hepatitis B [287]. Besides this, another study aimed to develop a starch hydrogel-based oral vaccine against Edwardsiellosis, and the results reveal it is an effective oral vaccine and can be used in aquaculture [288].

### 5.5. Vaginal Drug Delivery

Vaginal drug delivery has been considered as an alternative route of drug administration, owing to its several advantages of avoiding gastrointestinal degradation, avoiding the hepatic first-pass effect, its large contact surface area, and its good drug permeability. This drug delivery route is mainly intended for treating several diseases, including vaginal infections, atrophic vaginitis, cervical cancer and contraception [289]. Traditional formulations for vaginal delivery, such as creams, solutions, capsules, gels and vaginal suppositories, are commercially available, but these dosage forms are limited due to drug leakage, drug instability, short residence time and poor drug release [290]. New dosage forms have been extensively studied for vaginal drug delivery, and hydrogels have gained great interest owing to their unique properties [291].

Vaginal infections are a public health problem because of their high prevalence in adult women, and they can be divided into bacterial, fungal and parasitic infections. Hydrogels have been investigated as vehicles for the vaginal drug delivery of effective therapeutics for treating bacterial vaginosis. For example, a hydrogel (based on pluronics) was designed to deliver metronidazole to the vaginal mucosa for the treatment of bacterial vaginosis [292]. Another study also developed a carbopol-based hydrogel for the vaginal delivery of metronidazole [293]. Another type of hydrogel prepared by polycarbophil and carbopol was designed to deliver benzoyl peroxide vaginally to act against gardnerella vaginalis, indicating that a low concentration of drug-loaded hydrogel (0.01%) significantly inhibited the growth of microbes [294]. An HPMC and gellan gum hydrogel loaded with clindamycin was proposed to prolong drug residence time for use against vaginal mucosa [295]. In addition, a pH-responsive alginate hydrogel was prepared to improve the controlled release of polymyxin B via the vaginal route [296].

For treating fungal infections, hydrogels have been proposed as an alternative therapeutic. Amphotericin B was entrapped in cyclodextrins and then dispersed in a thermos-responsive hydrogel composed of pluronic [297]. This hydrogel was confirmed to constitute a promising system for the vaginal delivery of amphotericin B. Similarly, a thermos-responsive hydrogel containing amphotericin B was prepared from Poloxamer for treating vaginal fungal infections [298]. A hydrogel composed of Poloxamer has been developed to deliver clotrimazole via the vaginal route [299]. As for parasitic vaginal infections, a thermos-responsive chitosan hydrogel containing auranofin encapsulated in PLGA nanoparticles was proposed [300]. The results show that the drug retention time of the hydrogel in the vaginal mucosa exceeded 6 h, which significantly increased the concentration of the drug in the target tissue. Interestingly, a drug-free thermos-sensitive hydrogel (based on pluronic), loaded with mucoadhesive poly (isobutylcyanoacrylate) nanoparticles coated with chitosan and thiolated chitosan, was observed to show strong anti-Trichomonas vaginalis activity [301].

Additionally, chitosan ascorbate nanoparticles loaded with amoxicillin trihydrate were developed for the delivery of antibiotic drugs in the treatment of atrophic vaginitis [302]. Besides this, hydrogels have been extensively studied for the treatment of cervical cancer via the vaginal route. For example, amino group-functionalized imatinib nanocrystals were prepared and incorporated into thermos-responsive hydrogels (based on pluronic) for treating cervical cancer [303]. This hydrogel-based delivery system prolonged the drug retention time in the vaginal mucosa, and improved the inhibitory effect on tumor growth. Another study reported that a composite hydrogel consisting of chitosan and polyvinyl alcohol was prepared by chemical cross-linking, and was used as a vehicle for doxazocin, which significantly decreased angiogenesis in in vivo anti-cancer experiments [304]. The efficacy of a carboplatin-loaded Poloxamer hydrogel was evaluated for preventing the local recurrence of cervical cancer after surgery [305]. The study showed that the proposed Poloxamer hydrogel had great efficacy and systemic safety.

### 5.6. Rectal Drug Delivery

Rectal drug delivery is a noninvasive drug administration method that allows for both local and systemic effects. Traditional rectal dosage forms have mainly been used for their local effects, such as the delivery of laxatives, the administration of antipyretics and the treatment of hemorrhoids [306]. However, these dosage forms usually make patients have a foreign body sensation, and feel uncomfortable. Therefore, the development of novel rectal delivery systems is of great interest. Several novel rectal drug delivery systems, such as stimuli-responsive hydrogels, hollow-type suppositories, and nanoparticle systems incorporated into an appropriate vehicle, have been developed. Stimuli-responsive hydrogels, especially thermos-responsive hydrogels, have been used to deliver various drugs (such as anti-cancer, analgesic and antihypertensive drugs) via the rectal route because of their benefits, such as avoiding drug leakage out of the rectum, and precise dosing [307].

The use of anti-cancer drugs is commonly limited by the toxicity of the drugs, and rectal drug delivery can minimize this toxicity. 5-Fluorouracil, a widely used anti-cancer drug, was encapsulated in a thermos-responsive hydrogel for the treatment of colorectal cancer [308]. Docetaxel has also been loaded in a thermo-responsive hydrogel, the rheological properties of which were evaluated systemically [309]. A hydrogel containing epirubicin was administered to the rectums of rats, and the results show that this hydrogel was easily administered without leakage and retained in the rectum for at least 12 h [310]. Additionally, irinotecan [311], oxaliplatin [312], topotecan [313], regorafenib [314] and clotrimazole [315] have also been loaded into thermo-responsive hydrogels for the treatment of cancer via the rectal drug delivery route. It is worth mentioning that the rectal drug delivery of anti-cancer drugs may help to reduce hepatotoxicity when compared to oral drug delivery [315].

Analgesic drugs (also known as painkillers or pain relievers) are compounds that are used to relieve pain. These drugs are commonly administrated by oral or transdermal drug delivery systems. However, some drugs cannot be delivered via this route because of the properties of the drugs, including inappropriate hydrophilic/lipophilic properties, high first-pass effects and severe side effects. Therefore, these kinds of drugs have also been encapsulated in hydrogels and delivered by rectal drug delivery. For example, a thermos-responsive hydrogel loaded with acetaminophen was prepared and delivered into a rat rectum without difficulty or leakage, and kept in place for at least 6 h [316]. A thermos-responsive hydrogel prepared from Poloxamer was developed as a new carrier for the rectal drug delivery of nimesulide, resulting in significantly higher serum concentrations of drug when compared to the solid suppository [317]. More recently, tolmetin sodium (a nonsteroidal anti-inflammatory drug) was encapsulated in a thermo-responsive hydrogel composed of Poloxamer for rectal drug delivery so as to enhance patient compliance and avoid side effects [318].

Antihypertensive drugs are delivered mainly by the oral route for the treatment of hypertension. It has been reported that antihypertensive drugs encapsulated in hydrogels can also be administrated by rectal drug delivery, which may improve the pharmacokinetics of the drugs. For instance, propranolol, a beta-blocker, has been proven to achieve higher bioavailability via rectal drug delivery than via oral drug delivery [319]. Therefore, propranolol was encapsulated in various hydrogels for rectal drug delivery, and it was observed that sodium alginate and sodium polycarbonate displayed the greatest degrees of mucus adhesion and the lowest intrarectal migration, resulting in the maximum bioavailability of propranolol (84.7% and 82.3%, respectively) [320]. Besides this, a thermo-responsive hydrogel was prepared to deliver diltiazem hydrochloride (a calcium channel blocker) to the rectal mucosa with enhanced bioavailability of the drug [321].

### 5.7. Transdermal Drug Delivery

Transdermal drug delivery is an alternative to the oral and injection routes, and can deliver drugs across the skin to have local or systemic effects. It offers several advantages over other administration routes, including excellent patient compliance, noninvasive administration, and the avoidance of gastrointestinal degradation and the first-pass effect. However, the application of transdermal drug delivery is limited, because the corneum stratum of the skin blocks the efficient penetration of drugs. Many strategies, such as chemical enhancers and physical enhancement technologies, have been explored to enhance the capacity for drug penetration. Hydrogels have been used to increase drug penetration across the skin because of their hydration effects on the skin. The hydrogels used for transdermal drug delivery can be divided into two categories: common hydrogels and microneedle-based hydrogels.

Common hydrogels used as drug vehicles are directly applied on the skin (usually as a patch), and the drugs penetrate the skin mainly by diffusion. For example, an electrically responsive hydrogel was prepared using a polyacrylamide-grafted–pectin copolymer for the transdermal delivery of rivastigmine, which is a cholinesterase inhibitor used for the treatment of Alzheimer’s disease [322]. The permeation rate of rivastigmine was improved in the presence of an electric stimulus. A microemulsion-based hydrogel was designed as a carrier for the transdermal drug delivery of the total flavones of Arisaematis rhizoma, and the skin permeability of the drug was improved [323]. Acetylsalicylic acid was loaded into a modified chitosan hydrogel and delivered by the transdermal route to treat cardiovascular system diseases [324]. A novel hydrogel with high stretching and toughness properties has been used to encapsulate drugs including nicotine, lidocaine hydrochloride, diltiazem hydrochloride and diclofenac sodium for transdermal delivery [325].

Microneedles are needle-like micro-structures, usually composed of hundreds of needles in an array, with the length of each needle ranging from 25 to 1000 μm. Microneedles fabricated from hydrogels are considered as the newest type, named hydrogel-forming microneedles. This kind of microneedle has been used for transdermal drug delivery by adding drugs to polymer structures during the manufacturing or loading of drugs into separate reservoirs, and attaching them to the tops of the microneedles. For example, hydrogel-forming microneedle arrays encapsulating esketamine were fabricated to penetrate the outer layer of the skin, and thus enhance the drug penetration rate [326]. A novel hydrogel-forming microneedle array was used for the transdermal delivery of tuberculosis drugs, including rifampicin, isoniazid, pyrazinamide and ethambutol. These drugs were incorporated into various types of drug reservoirs, and further integrated with hydrogel-forming microneedle arrays for enhanced drug delivery [327]. Additionally, a poly (N-isopropylacrylamide)-based hydrogel was used for the transdermal drug delivery of insulin, which can be controllably released to regulate blood glucose levels in streptozotocin-induced diabetic mice [328].

### 5.8. Subcutaneous and Intramuscular (Injection) Drug Delivery

Administration by injection (parenteral administration) mainly includes subcutaneous (under the skin) and intramuscular (in a muscle) drug delivery [329]. Subcutaneous drug delivery refers to the injection of drugs into the subcutaneous layer of an individual (the layer between the skin and the muscle), and it is commonly used with diabetes and cancer patients [330]. This type of drug delivery approach has been proven to be safe, well tolerated, and effective, and is the first choice for healthcare providers and patients, especially for the delivery of many protein drugs [330]. However, the dose of subcutaneous drug delivery is limited to 2 mL. Therefore, intramuscular drug delivery is preferred to the subcutaneous route when larger volumes of drug are required. It is also known as an intramuscular injection, wherein the drug enters deep into the muscle tissue, through the dermis and subcutaneous tissue, and into the deepest layer of the muscle, where the strong blood supply allows rapid and full absorption [331]. Hydrogels have been used as vehicles for both subcutaneous and intramuscular drug delivery.

For example, a hyaluronic acid-based hydrogel was loaded with donepezil for subcutaneous injection [332]. Donepezil can be released from the hydrogel in a sustained manner after subcutaneous injection. Another form of subcutaneous injection of a hyaluronic acid hydrogel was proposed by the same research group [333]. The hydrogel was hybridized with a microstructural lipid carrier and human serum albumin, which was used for the sustained release of donepezil and to reduce the initial burst release. An injectable peptide-based hydrogel was developed as a controlled drug delivery system for the subcutaneous delivery of opioids (morphine and 14-methoxymetopon) [334]. The applicability of the peptide hydrogel was verified in vivo by tail flick tests in mice, and a sustained antinociceptive effect was obtained after the subcutaneous injection of the hydrogel.

Additionally, several anti-cancer drugs, such as docetaxel, paclitaxel, doxorubicin, tamoxifen, herceptin and avastin, have all been encapsulated in various hydrogels for treating cancers such as lung, breast, liver, cervical and colorectal cancer, which have been reviewed by Donnelly et al. [335]. Moreover, hydrogels (based on peptide) have been demonstrated to be potent vaccine adjuvants, and are thus used as the vehicles for vaccines. For example, a composite hydrogel prepared by physically cross-linking was used to deliver cancer preventive vaccines subcutaneously to mice, and a vaccine-loaded hydrogel can stay in the body for more than 17 weeks to ensure continuous anti-cancer immunity [336].

As for intramuscular drug delivery, stimuli-responsive hydrogels are of great interest for researchers as a delivery system for proteins and other hydrophilic macromolecular drugs. For instance, a thermos-responsive hydrogel composed of Poloxamer was developed and evaluated for the sustained delivery of hydrophilic macromolecules by the intramuscular drug delivery route [337]. A pH-responsive phosphorylated chitosan hydrogel was prepared for loading with ovalbumin antigen, and intramuscularly injected into mice [338]. The results show that the use of a hydrogel as a vaccine delivery system can significantly improve the level of antigen-specific immune response. Similarly, an ovalbumin antigen encapsulated in a hydrogel was released in a significantly more sustainable way, and resulted in a more potent antigen-specific IgG immune response [339].

Besides this, many chemical drugs have also been encapsulated in hydrogels for intramuscular drug delivery. A thermos-responsive hydrogel containing doxorubicin was prepared with Poloxamer and hydrochloric acid, and administrated intramuscularly to rats [340]. It was observed that the plasma concentrations of the drug were maintained for 60 hours, and yielded about 5-fold greater AUC (areas under the curve) compared to the doxorubicin solution. Another study reported that a novel thermos-responsive hydrogel containing piroxicam was delivered by intramuscular drug delivery [341]. Moreover, the same group proposed the same hydrogel for the intramuscular drug delivery of irinotecan, for the treatment of cancers [342].

## 6. Conclusions

In this review, we summarize the classification of hydrogel on the basis of source/origin, polymeric composition, configuration/structure, response to stimuli, durability, and network charge. The fabrication strategies of hydrogels, including magnetic hydrogels, are explained in detail. Generally, hydrogels are prepared by a cross-linking method using physical cross-linking or chemical cross-linking, while magnetic hydrogels are prepared by in situ methods, blending methods or grafting-onto methods. In addition, the applications of hydrogels in various fields, including wound healing, tissue engineering, water processing, agriculture, food, sensors, firefighting, and other applications (absorbent hygiene products and contact lenses), are discussed. Hydrogels have been widely used due to their advantages over other types of biomaterials, including good swelling behavior, suitable mechanical strength, increased biocompatibility, tunable biodegradability, and low toxicity. Besides this, they have also been broadly applied in the field of drug delivery. Recent advances in hydrogel preparation and its applications in the aforementioned are reviewed and discussed in full detail.

## Data Availability

Not applicable.
